# Cholesterol Levels and Statin Use in Patients With Coronary Heart Disease Treated in Primary Care Settings

**Published:** 2005-06-15

**Authors:** Patrick J O’Connor, Richard J Gray, Michael V Maciosek, Kelly M Fillbrandt, Terese A DeFor, Charles M Alexander, Thomas W Weiss, Steven M Teutsch

**Affiliations:** HealthPartners Research Foundation and HealthPartners Medical Group; HealthPartners Research Foundation and HealthPartners Medical Group, Minneapolis, Minn; HealthPartners Research Foundation and HealthPartners Medical Group, Minneapolis, Minn; HealthPartners Research Foundation and HealthPartners Medical Group, Minneapolis, Minn; HealthPartners Research Foundation and HealthPartners Medical Group, Minneapolis, Minn; HealthPartners Research Foundation and HealthPartners Medical Group, Minneapolis, Minn; HealthPartners Research Foundation and HealthPartners Medical Group, Minneapolis, Minn; Merck & Co, Inc, West Point, Pa

## Abstract

**Introduction:**

Therapy with 3-hydroxy-3-methylglutaryl coenzyme A (HMG-CoA) reductase inhibitors, or statins, has proven to be effective in the treatment of lipid disorders. However, statin therapy continues to be underused, even though statins are a relatively safe and well-tolerated class of agents. In this study, we assessed trends in lipid control in patients with heart disease who receive most of their health care in primary care clinics. The objective was to determine whether systems of care implemented within a large medical group are associated with improved treatment and control of dyslipidemia in a high-risk group of coronary heart disease patients.

**Methods:**

All adults with heart disease in a Minnesota medical group (N = 2947) were identified using diagnosis and procedure codes related to coronary heart disease (sensitivity = 0.85; positive predictive value = 0.89) in 1996. Study subjects were observed from 1995 to 1998. Subjects had a baseline and follow-up test for low-density lipoprotein cholesterol and high-density lipoprotein cholesterol. Changes between baseline and follow-up measurements and trends in the use of statins and other lipid-active agents among the study subjects were analyzed.

**Results:**

Among 1388 subjects with two or more eligible lipid measurements, mean low-density lipoprotein cholesterol improved from 137.6 mg/dL to 111.0 mg/dL (*P* < .001), and mean high-density lipoprotein cholesterol improved from 42.3 mg/dL to 46.3 mg/dL (*P* < .001). The percentage of patients with low-density lipoprotein cholesterol ≤100 mg/dL rose from 12.5% to 39.8% (*P* < .001), and the percentage with high-density lipoprotein cholesterol ≥40 mg/dL rose from 52.5% to 67.6% (*P* < .001). In multivariate models, statin use was identified as the main factor that contributed to the improvement in low-density lipoprotein cholesterol (*P* < .001). Men had greater decreases in low-density lipoprotein cholesterol than women after adjusting for other variables (*P* < .001). Statin use rose from 24.3% at baseline to 69.6% at follow-up. The statin discontinuation rate was 8.3% for baseline statin users and 12.2% for subjects who used statins at any time during the study period.

**Conclusion:**

Investment in better heart disease care for patients in primary care clinics led to major improvement in lipid control over 30 months, primarily due to increased statin use. Improvements in low-density lipoprotein cholesterol and high-density lipoprotein cholesterol were sufficient to substantially reduce risk of subsequent major cardiovascular events.

## Introduction

Clinical trials provide strong support for using 3-hydroxy-3-methylglutaryl coenzyme A (HMG-CoA) reductase inhibitor (statin) therapy in patients with coronary heart disease (CHD) (1-7). Statins are a relatively safe and well-tolerated class of agents that have proven to be effective in treating lipid disorders. Recent data suggest, however, that statin therapy remains underused in the treatment of lipid disorders, or dyslipidemia. Reasons for this underuse of statins include inadequate physician titration of medications to reach the goal level of low-density lipoprotein cholesterol (LDL-C) recommended by the National Cholesterol Education Program (NCEP) and inadequate long-term patient adherence to prescribed drug therapy (8-10).

In this study, we assessed trends in the use of statin therapy and changes in LDL-C levels in a well-defined population of adults with CHD receiving their care at a single large multispecialty medical group. During the 4-year study period, the medical group emphasized the importance of lipid control in CHD patients. Primary care physicians had unrestricted access to several statins through the medical group's drug formulary. Clinical guidelines for lipid control emphasized aggressive pharmacotherapy, and patients received messages on the importance of lipid control through periodic medical group publications sent to their homes. Our study objective was to determine whether systems of care implemented within a large medical group are associated with improved treatment and control of dyslipidemia in a high-risk group of CHD patients.

## Methods

### Study site

The study was conducted at HealthPartners Medical Group (HPMG), a large multispecialty group practice in Minnesota established in 1957 that in 1998 provided care to 220,000 patients insured by HealthPartners. Patients received clinical care at one of 18 primary care clinics staffed by internal medicine and family practice physicians. 

About 75% of CHD patients at HPMG have pharmaceutical benefits as part of their health insurance. Most patients with such benefits had a copayment of $10 to $15 for each 30-day supply of a prescription medication during the study period. Most patients without pharmaceutical benefits were aged 65 years or older and chose not to have such coverage because of the additional monthly premium required. There were no other out-of-pocket costs to plan members or disincentives to physicians for measuring serum lipids or prescribing lipid treatment as desired. The medical group formulary included unrestricted physician prescribing of statins. During the study period, lovastatin (Mevacor), fluvastatin (Lescol), pravastatin (Pravachol), simvastatin (Zocor), and atorvastatin (Lipitor), along with a wide selection of other lipid active agents — including gemfibrozil (Lopid), niacin preparations, and cholestyramine — were available for unrestricted use.

Local clinical practice guidelines for lipid screening and treatment were implemented in 1995 and updated annually through the Institute for Clinical Systems Improvement (ICSI), a collaborative health improvement organization organized and supported by HealthPartners and many other Minnesota health care organizations, including the Park Nicollet Clinic, Allina Medical Clinic, and the Mayo Clinic. ICSI lipid treatment guidelines (11) are similar to those of NCEP (12) and, at the time of the study, emphasized aggressive pharmacotherapy for patients with CHD to reduce LDL-C levels to <100 mg/dL.  

### Study subjects

Study subjects were adult patients identified as having CHD in 1996. A diagnosis of CHD was assigned to any patient meeting at least one of the following criteria in 1996: at least two diagnoses from among codes 410.xx–414.xx or 429.2 in the *International Classification of Diseases, Ninth Revision, Clinical Modification *(*ICD-9-CM*); at least one procedure from among codes 33510–33545 and 36822 in *Current Procedural Terminology* (*CPT*); or at least one procedure from among codes 36.0–36.29 and 36.9–36.99 in the *International Classification of Diseases, Ninth Revision* (*ICD-9*). This process of identifying study subjects has been formally assessed and has an estimated sensitivity of 0.85, specificity of 0.99, and positive predictive value of 0.89 (13). Each eligible study subject was aged 19 years or older on January 1, 1996, and was continuously enrolled for care at HPMG during the 1996 calendar year. This process identified 2947 eligible patients with heart disease in 1996. The study was reviewed, approved, and monitored by the HealthPartners Institutional Review Board.

### Definition and measurement of variables

Trends in lipid control were necessarily based on change in LDL-C levels from a baseline test to a follow-up test. The baseline test was defined as the first LDL-C test done on or after January 1, 1995. The follow-up test was defined as the most recent LDL-C test done before December 31, 1998. Furthermore, the follow-up LDL-C test must have been done at least 365 days after the baseline measurement. Thus, for eligible baseline and follow-up data, a study subject must have had at least two LDL-C measures at least 365 days apart between January 1, 1995, and December 31, 1998. Of 2947 eligible study subjects aged 19 years or older with CHD, a total of 1388 (47%) had two qualifying LDL-C measurements recorded during the specified time period. Among the 1559 without two qualifying LDL-C measures, 155 died or disenrolled by December 31, 1998. Changes in high-density lipoprotein cholesterol (HDL-C) were also measured using the same definition of test dates and period between tests.

To explore associations between changes in LDL-C and statin use from administrative data, we defined a second subpopulation. We analyzed data on individuals with two qualifying LDL-C measurements and pharmacy coverage provided by HealthPartners. There were 1038 in this subpopulation (75% of study subjects with two LDL-C measurements). Pharmacy coverage was defined as present from baseline to follow-up LDL-C tests, allowing for a gap in coverage of up to 60 days during that period.

All lipid tests during the study period were performed at a single accredited clinical chemistry laboratory using standard assay methods (14-16) that did not change during the study period. Values of LDL-C were calculated in milligrams per deciliter (mg/dL) using the Friedewald formula (17). The fasting period prior to taking the blood sample was recorded along with each test result; only LDL-C values drawn after a minimum 12-hour fast were used in this study. Levels of HDL-C directly measured in mg/dL using a standard assay were also obtained from automated laboratory databases. Changes in LDL-C or HDL-C from baseline to follow-up were calculated as the follow-up LDL-C (or HDL-C) minus the baseline level. A negative number thus represents improvement in LDL-C over time, and a positive number represents improvement in HDL-C over time.

All identified CHD patients were surveyed by mail with a telephone follow-up (18) in 1998 to assess demographic variables, such as age, sex, height, weight, educational level, smoking status, and aspirin use; 2122 (72%) of the 2947 study-eligible members returned surveys with complete responses on these variables.

Administrative data were used to identify patients with CHD and their age, sex, pharmacy coverage, filled prescriptions for statins, and filled prescriptions for other cholesterol-acting agents. For all patients with pharmacy coverage, pharmacy databases provided name of medication, dose per tablet, number of tablets dispensed, and the dispense date. For multivariate analysis, we measured statin use as the portion of the days between the baseline and follow-up LDL-C measures for which the subject had a filled statin prescription. We measured the use of other cholesterol-acting agents ("other use") in the same manner. These agents included fibrates, resins, nicotinic acid, probucol, and oral estrogens. Over-the-counter niacin formulations were not tracked on the pharmacy database. Lipid tests and results were available from electronic databases, as was information on health plan enrollment.

### Plan of analysis

Initial analysis was done to assess the distributions of baseline LDL-C, follow-up LDL-C, change in LDL-C, and other lipid measures. Differences in demographic variables between participants with and without two qualifying LDL-C measurements were analyzed using two-sample *t* tests for continuous variables and chi-square tests for count variables. The Satterthwaite adjustment for unequal variances in two-sample tests was used when appropriate. Changes over time in continuous variables were analyzed using the single-sample *t* test of the difference between baseline and follow-up for continuous variables and the McNemar test for count variables. Multivariate linear regression was used to estimate relationships between the difference in baseline and follow-up LDL-C as well as the difference in HDL-C as the key dependent variables. In all analyses, a two-tailed α of 0.05 was used to test for significant associations.

## Results

The overall population of individuals with at least two qualifying LDL-C measures (n = 1388) is described in Table 1. Although baseline and follow-up lipid measures were required to be at least 365 days apart, the median time between the measures was 917 days, or 2.5 years.

The mean age of study subjects with two qualifying LDL-C measures was 64.9 years; 66.6% were male, and 24.1% had a college degree. Current smoking was reported by 7.1%, and 59.4% indicated a prior history of smoking. Regular aspirin use was reported by 82.9%. Mean body mass index (BMI) was 27.1 kg/m^2^, and 24.3% were identified as having diabetes at baseline. There were statistically significant differences between study-eligible members with and without two qualifying LDL-C measurements for all variables other than BMI and baseline diabetes.

Table 2 describes LDL-C and HDL-C measures at baseline and at follow-up. Data are presented for all patients with qualifying LDL-C measurements (n = 1388), for patients with baseline LDL-C >100 mg/dL (n = 1214), and for patients with baseline LDL-C >130 mg/dL (n = 781). For all patients with qualifying measurements, there was a significant decline in mean LDL-C, from 137.6 mg/dL at baseline to 111.0 mg/dL at follow-up, a change of -26.6 mg/dL (*P* < .001). Mean HDL-C also improved, with an increase from 42.3 mg/dL at baseline to 46.3 mg/dL at follow-up, a change of +4.0 mg/dL (*P* < .001). For the subgroup with baseline LDL-C >100 mg/dL, mean LDL-C decreased from 144.7 mg/dL at baseline to 113.5 mg/dL at follow-up, a change of -31.2 mg/dL (*P* < .001). Mean HDL-C in this subgroup increased from 42.8 mg/dL to 46.6 mg/dL, a change of +3.8 mg/dL (*P* = .008). For the subgroup with baseline LDL-C >130 mg/dL, the change in mean LDL-C was -42.5 mg/dL (*P* < .001), and the change in HDL-C was +3.5 mg/dL (*P* = .02). The percentage of patients in the study with LDL-C ≤100 mg/dL rose from 12.5% to 39.8% (*P* < .001), and the percentage of patients with HDL-C ≥40 mg/dL rose from 52.5% to 67.6% (*P* < .001).

Table 3 shows the proportion of patients receiving statins (among the 1038 subjects with pharmacy coverage and two qualifying LDL-C measures) at both baseline and follow-up and the subsequent discontinuation rate more than 1 year later in two groups of statin users: patients who used statins at baseline and patients who used statins at any time during the study period. Statin use among all study subjects in this group was 24.3% at baseline and 69.6% at follow-up. In those with baseline LDL-C >100 mg/dL, statin use was 21.0% at baseline and 70.4% at follow-up. In those with baseline LDL-C >130 mg/dL, statin use was 16.7% at baseline and 74.2% at follow-up. Statin discontinuation rates were 8.3% for baseline statin users, 9.5% for baseline users whose baseline LDL-C was >100 mg/dL, and 4.1% for baseline statin users whose baseline LDL-C was >130 mg/dL. For subjects who used statins at any time during the study period, the discontinuation rate was 12.2%.

Table 4 reports the multivariate analysis of LDL-C and HDL-C changes and statin use for the 1038 study-eligible members with pharmacy coverage. The model presented in Table 4 includes administrative data only. The model presented in Table 5 adds survey data and is limited to the 804 individuals included in the previous model who returned surveys with completed responses on variables of interest. Residual plots showed neither excessive nonnormality in the residuals nor heteroskedasticity.

In the models of Table 4, baseline LDL-C, sex, and statin use are significant predictors of decreases in LDL-C. Table 5 shows that use of statins for the entire period between baseline and follow-up measures (i.e., statin use = 1.0) was associated with an additional 17 mg/dL decrease in LDL-C compared with no statin use. On average, men experience an additional 7 mg/dL decrease in LDL-C compared with women. Only baseline HDL-C and sex are significantly related to changes in HDL-C. On average, women experienced a 1.7 mg/dL greater increase in HDL than did men. Among the variables collected from surveys (Table 5), aspirin use was associated with a statistically significant (6 mg/dL) decrease in LDL-C, and a 1-point decrease in BMI was associated with a 0.29 mg/dL improvement in HDL-C.

## Discussion

We analyzed trends in LDL-C and HDL-C levels in patients with diagnosed heart disease having two LDL-C measurements in a large medical group. Statin use was analyzed for the subgroup of 1038 (75%) with pharmacy coverage. At baseline, all patients had established CHD and hence would have had an NCEP-established goal of LDL-C <100 mg/dL at the time of the study. However, at baseline only 24.3% were receiving statin therapy, and only 12.5% had LDL-C of ≤100 mg/dL. After a mean follow-up period of 2.5 years, mean LDL-C and HDL-C levels had significantly improved in the entire cohort, as well as in subgroups stratified by baseline LDL-C of >130 mg/dL and >100mg/dL. There was a corresponding marked increase in statin use during the follow-up period, with a significant increase in patients achieving the study goal of LDL-C ≤100 mg/dL (12.5% at baseline vs 39.8% at follow-up, *P* < .001).

These data document a significant improvement in the proportion of CHD patients treated with statins and reaching their NCEP-established LDL-C goal in the late 1990s (19-21). Our results favorably compare with contemporaneous data collected on the general Minnesota population, which showed worsening cholesterol levels in the 1990s (8). Results also favorably compare with the survey of 48,586 individuals with coronary artery disease from practices throughout the United States, chosen for their frequent use of cholesterol-active medications, in which 44% had annual testing of LDL-C. Of those who had annual testing, only 25% reached the target LDL-C of ≤100 mg/dL, and only 39% were taking lipid lowering therapy (9).

The Lipid Treatment Assessment Project (L-TAP) was a comprehensive survey of lipid therapy prescribing habits and lipid results conducted in five regions of the United States and drawn from a group of primary care physicians, also targeted because they wrote large numbers of prescriptions for lipid-active drugs (10). The L-TAP study demonstrated that in a cohort of 1460 patients with CHD, only 18% attained the NCEP-established goal of LDL-C <100 mg/dL, compared with 39.8% in our study who attained an LDL-C level of ≤100 mg/dL. This was despite the fact that 84.6% received treatment with lipid lowering agents, compared with 69.6% receiving statin therapy in our study (10).

While baseline LDL-C levels were being measured for this study (median date of October 12, 1995), a system-wide program of health-related goals was implemented at HPMG. Among these goals was a heart-health goal that called for a 25% reduction in CHD events within 4 years (22). The importance of lipid control in CHD patients was emphasized by medical group leaders in meetings with primary care physicians and certain subspecialty physicians, including cardiologists.

During the years of this study, systems of care were developed and deployed within the medical group to support both patient and provider attention to control of lipid disorders (22-26). Results of lipid testing were stored for electronic retrieval via computer. Lipid test results, with an explanation and recommendations, were mailed to patients. High-risk patient registries that included adults with CHD or diabetes and LDL-C test results or indicated a need to obtain an LDL-C test were provided to clinics and physicians as an aid for tracking, visit planning, and active outreach. One of the authors (RJG) gave a series of lectures at each medical group clinic about the use and benefits of lipid-lowering therapy. Also, a specialized program known as Lifestyle Management was added to the cardiac rehabilitation program. This program featured one-on-one sessions with nursing staff for patients and follow-up case management, as well as a direct review of each patient's care plan and progress by the medical director. During the study period, it also became commonplace to initiate statin therapy before hospital discharge following an acute cardiac event, such as a myocardial infarction. These programs, initiated to aggressively reduce cardiovascular events in high-risk patients during the study period, likely accounted for some of the observed improvement in lipid control. It is unlikely that secular trends accounted for all the improvement, because few other medical groups have achieved similar levels of lipid drug use (27) or lipid control (28), and lipid trends in Minnesota during these years were not improving (8).

Several factors limit the interpretation of the data presented here. First, misclassification of CHD status is a possibility; however, we used a validated method for heart disease identification with an estimated sensitivity of 0.85 and a positive predictive value of 0.89 (13). Second, the study was limited to one large medical group in Minnesota. Studies in a variety of other settings may be needed to replicate and extend the findings reported here. Finally, the requirement that each patient have at least two LDL-C measurements over an average 30-month period may have identified a population of patients having more active management of dyslipidemia. However, this selection strategy was essential, given our intent to assess trends of LDL-C and statin use over time. We were unable to compare patients with and without qualifying LDL-C measures in relation to baseline LDL-C levels or statin use. We observed at least one LDL-C in only a portion of those without two LDL-C measurements, and the majority of the single LDL-C measures that exist were observed near the end of the 4-year study period. Therefore, the available LDL-C measures for the group excluded from the analysis do not provide an analogous baseline LDL-C measure. Likewise, our measure of statin use is tied to LDL-C baseline and follow-up measures and could not be computed for participants without two qualifying measures.

We conclude that substantial improvement in LDL-C and HDL-C control occurred in adults with CHD at this practice site during the late 1990s. The main cause of the improvement was a dramatic increase in statin use, which was significantly related to LDL-C change. However, other factors contributed to improved lipid control in this population, including coordination of use of clinical guidelines among clinics, use of high-risk patient registries, and use of automated monitoring and prioritizing of patients for special attention. As LDL-C goals become more stringent (3,4,6), the application of effective outpatient chronic disease care strategies such as registries, active outreach, visit planning, and coordination of care across sites will increase in importance. These data show that primary care clinics and providers are capable of dramatic improvements in care over a short period and suggest that resources invested to improve outpatient care can rapidly return a sizeable clinical return on investment.

## Figures and Tables

**Table 1:** Characteristics of Adult Health Plan Members With Coronary Heart Disease (N = 2947), by LDL-C Test Status, 1996^a^

**Characteristic^b^**	**Members With Two Qualifying LDL-C Measurements (n = 1388)**	**Members Without Two Qualifying LDL-C Measurements (n = 1559)**	***P***
Mean age (years)	64.9	68.4	<.001
Male	66.6	52.3	<.001
Baseline diabetes	24.3	25.0	.67
College degree (2307)	24.1	18.3	.001
Current smokers (2193)	7.1	10.5	.005
Former smokers (2193)	59.4	50.7	<.001
Regular aspirin use (2201)	82.9	65.8	<.001
Mean BMI^c^ (2097)	27.1	26.8	.15
BMI >25 (2097)	72.2	63.9	<.001
BMI >30 (2097)	24.5	26.0	.43

a Values are percentages unless otherwise indicated. LDL-C indicates low-density lipoprotein cholesterol.

b Sample size for each survey variable when different from N shown in parentheses.

c BMI indicates body mass index (kg/m^2^).

**Table 2:** Change in LDL-C and HDL-C Among Adult Health Plan Members With Two Qualifying LDL-C Measurements, 1995–1998^a^

**Characteristic**	**Baseline**	**Follow-up**	**Change**	***P***

**All subjects (n=1388)**				

LDL-C (mean mg/dL)	137.6	111.0	−26.6	<.001
HDL-C (mean mg/dL)	42.3	46.3	+4.0	<.001
LDL-C ≤100 mg/dL (% subjects)	12.5	39.8	+27.3	<.001
HDL-C ≥40 mg/dL (% subjects)	52.5	67.6	+15.1	<.001

**Subjects with baseline LDL-C >100 mg/dL (n=1214)**				

LDL-C (mean mg/dL)	144.7	113.5	−31.2	<.001
HDL-C (mean mg/dL)	42.8	46.6	+3.8	.008
LDL-C ≤100 mg/dL (% subjects)	0	36.2	+36.2	NA
HDL-C ≥40 mg/dL (% subjects)	54.5	68.4	+13.9	<.001

**Subjects with baseline LDL-C >130 mg/dL (n=781)**				

LDL-C (mean mg/dL)	160.8	118.3	−42.5	<.001
HDL-C (mean mg/dL)	43.8	47.3	+3.5	.02
LDL-C ≤100 mg/dL (% subjects)	0	31.4	+31.4	NA
HDL-C ≥40 mg/dL (% subjects)	58.5	72.6	+14.1	<.001

a LDL-C indicates low-density lipoprotein cholesterol; HDL-C, high-density lipoprotein cholesterol; and NA, not applicable.

**Table 3:** Statin Use Among Adult Health Plan Members With Coronary Heart Disease, Two Qualifying LDL-C Measurements, and Pharmacy Coverage (n = 1038), 1995–1998^a^

	**Statin Use**				**Statin Discontinuation Rates Before Follow-up**	

	**Baseline (%)**	**Follow-up (%)**	**Change (%) **	***P***	**Baseline Users (%)**	**Users During Study Period (%)**
All subjects (n=1038)	24.3	69.6	+45.3	<.001	8.3	12.2
Baseline LDL-C >100 mg/dL (n=899)	21.0	70.4	+49.4	<.001	9.5	12.8
Baseline LDL-C >130 mg/dL (n=563)	16.7	74.2	+57.5	<.001	4.1	12.3

a LDL-C indicates low-density lipoprotein cholesterol.

**Table 4:** Multivariate Analysis of Changes in LDL-C and HDL-C Among Adult Health Plan Members (n = 1038) in Relation to Statin Use and Baseline Lipid Levels, With Adjustment for Demographics^a^

**Variable**	**Change in LDL-C^b^**			**Change in HDL-C^b^**		

	**Coefficient Estimate**	**Standard Error**	***P***	**Coefficient Estimate**	**Standard Error**	***P***
Intercept	90.38	7.465	<.001	14.53	2.013	<.001
Baseline LDL-C	−0.69	0.025	<.001	NA	NA	NA
Baseline HDL-C	NA	NA	NA	−0.28	0.022	<.001
Days between baseline and follow-up	−0.0015	0.003	.62	0.0000029	0.0008647	.997
Statin use	−17.18	2.238	<.001	0.67	0.639	.29
Use of other cholesterol-acting drugs	−5.32	2.881	.07	1.48	0.8295	.08
Age	−0.10	0.087	.25	0.027	0.025	.28
Male	−7.36	2.056	<.001	−1.67	0.625	.007
Adjusted R^2^	0.459	NA	NA	0.142	NA	NA
F value	147.9	NA	<.001	29.6	NA	<.001

a LDL-C indicates low-density lipoprotein cholesterol; HDL-C, high-density lipoprotein cholesterol. These health plan members had two qualifying LDL-C measures plus pharmacy coverage. This model includes administrative data only.

b Changes in LDL-C and HDL-C are measured as the follow-up value minus the baseline value. NA indicates not applicable.

**Table 5:** Multivariate Analysis of Changes in LDL-C and HDL-C in Relation to Statin Use and Baseline Lipid Levels Among Adult Health Plan Members (n = 804), With Adjustment for Demographics and Selected Cardiovascular Disease Risk Factors^a^

**Variable**	**Change in LDL-C^b^**			**Change in HDL-C^b^**		

	**Coefficient Estimate**	**Standard Error**	***P***	**Coefficient Estimate**	**Standard Error**	***P***
Intercept	88.73	12.351	<.001	25.19	3.462	<.001
Baseline LDL-C	−0.69	0.028	<.001	NA	NA	NA
Baseline HDL-C	NA	NA	NA	−0.31	0.025	<.001
Days between baseline and follow-up	−0.0026	0.0034	.45	0.00040	0.00096	.67
Statin use	−17.08	2.541	<.001	1.44	0.707	.04
Use of other cholesterol-acting drugs	−4.04	3.273	.22	1.47	0.915	.11
Age	−0.025	0.109	.81	−0.00057	0.0304	.99
Male	−7.32	2.490	.003	−2.51	0.751	<.001
Current smoker	5.87	3.851	.13	0.87	1.077	.42
Former smoker	1.22	2.191	.58	0.99	0.613	.11
BMI^c^	−0.058	0.213	.79	−0.29	0.060	<.001
Regular aspirin use	−5.91	2.640	.03	0.43	0.738	.56
High school diploma	4.33	2.328	.06	−0.58	0.652	.37
College degree	1.94	2.568	.45	0.43	0.600	.55
Adjusted R^2^	0.452	NA	NA	0.174	NA	NA
F value	56.2	NA	<.001	15.1	NA	<.001

a LDL-C indicates low-density lipoprotein cholesterol; HDL-C, high-density lipoprotein cholesterol. These health plan members had two qualifying LDL-C measures plus pharmacy coverage, and they completed health surveys. This model includes administrative data as well as subject survey data.

b Changes in LDL-C and HDL-C are measured as the follow-up value minus the baseline value. NA indicates not applicable.

c BMI indicates body mass index (kg/m^2^).
